# Arguments for a comet as cause of the Hopewell airburst are unsubstantiated

**DOI:** 10.1038/s41598-022-16211-5

**Published:** 2022-07-15

**Authors:** Ralph Neuhäuser, Dagmar L. Neuhäuser

**Affiliations:** 1 Astrophysikalisches Institut, Universität Jena, Schillergässchen 2-3, 07745 Jena, Germany; 2 Unaffiliated/independent scholar, Meran/o, Alto Adige, Italy

**Keywords:** Planetary science, Astronomy and planetary science

**arising from:** K. B. Tankersley et al.; *Scientific Reports* 10.1038/s41598-022-05758-y (2022).

## Introduction

It was claimed that Hopewell habitation surface (Ohio, USA) was burned in a catastrophic event between CE 252 and 383, dated by radiocarbon^1^, being caused by an airbursting *comet* entering the Earth atmosphere. However, the material evidence (meteorites, Fe and Si-rich microspherules, positive Ir and Pt anomalies, burned carbon-rich habitation surfaces, blanket of ejecta, lack of crater)^1^ does not speak for a comet compared to an asteroid, as in Tunguska^2^ and other cases^3^; it neither proofs an airburst at that site nor that time, c.f. the Brenham meteorite that fell 20 kyr ago in Kansas^4^. Given those uncertainties, the three additional arguments given for a comet^1^ are essential; we focus on those.

(i) Increased risk due to comets? Tankersley et al.: “the event occurred between 252 and 383 CE, a time when 69 near-Earth comets were documented”^1^, and with reference to Tsu (1934): “between 1800 and 1431 years ago (220 and 589 CE), Chinese astronomers documented 69 near-Earth comets (< 1.3 au and a period of revolution < 200 years), including Haley’s, which came within 0.09 au of earth in 374 CE”^1^.

Indeed, Tsu has: “A.D. 220 to 589, there were 69 comets and 13 new stars recorded”^5^, but neither the texts nor the years of those objects are given. There is no evidence that all the presumable 69 comets CE 220–589 occurred CE 252–383 nor that they were near-Earth comets. More recent works^6,7^ give ca. 40 transients observed in East Asia CE 252–383 including other objects like nova/supernovae and atmospheric phenomena. Kronk^8^ lists up to 41 comet candidates CE 252–383 worldwide, none of them with a known orbit except 1P/Halley.

The closest approach of 1P/Halley to Earth in the last two millennia^9^ was in CE 837, not CE 374. For all perihelia before CE 837, the orbital solutions are less certain due to that close approach^9^. The orbit for CE 760 was recently solved with historical observations only^10^. Backward extrapolations are also problematic for comet 109P/Swift-Tuttle^10^, e.g. the observation of CE 188 July 28 from China of “a `guest star’ as large as a vessel with a capacity of 3 pints”^6^ is not typical for comets, e.g. no duration is given, it could just be a fireball^8^.

In sum, the claim that “human communities … were at a heightened risk … by a comet airburst event”^1^ in those centuries is unsubstantiated: Evidence for *higher* than normal comet frequencies or *more* near-Earth comets was not presented.

(ii) Is the “comet-shaped earthwork” related to the airburst? A sketch of the now overbuild Milford earthwork (Ohio), looking somewhat comet-shaped from above, is presented as archaeological evidence in favor of a cometary airburst (Figure 25, part B in ref.^1^). Irrespective of whether this earthwork should depict the comet outside or inside the Earth atmosphere: If a comet in today’s sense would have entered the Earth atmosphere to cause the airburst around CE 252–383, this comet were visible close to Earth in the preceding nights (or even weeks), bright and long-tailed—it would have been noticed by night-time observers all over the hemisphere. In the Classical Chinese text corpus, the time CE 252–383 with the Three Kingdom (CE 220–265) and Jin dynasty (CE 265–420) is well-documented^5–9^, and during transition periods from a late unstable dynasty to a new one, court astronomers/astrologers have kept a particularly close watch. The archives from Europe as well as West and East Asia do not include an extraordinary record on a bright, possibly broken-up comet that is *also* reported to have disappeared when still large. As all 1P/Halley perihelia of the last two millennia were noticed^5–9^, comet records are quite complete, in particular for bright and long-tailed comets. Also earlier, comet impacts were incorrectly suggested for cosmic events with signatures on Earth^3,11–13^.

The "comet-shaped earthwork" is only one feature in a larger structure, one would need to explain all other parts also in connection to an airbursting comet (Figure 25, part B^1^). Other, more extended illustrations^14^ show another circle + road-arrangement in almost opposite direction. The stylized re-drawing (Figure 25 part A in ref.^1^) omits another path connecting the circle of the "comet-shaped earthwork" with another large artificial structure. Also, other possible explanations for the conically shaped path leading to an elevated circle should be considered, e.g. a procession path. If the impact contributed to the Hopewell decline^1^, it appears unlikely that such a large structure was built after the event (and meteorites were apparently traded by the Hopewell before and afterwards, explaining the heterogeneity of their meteorites^1^). Even if the earthwork should mirror the airburst itself, the so-called "comet-shaped" structure does not provide evidence for a *cometary* origin: hand drawings by contemporary witnesses of the Tunguska event are not dissimilar in form^15^, but the cause was likely an asteroid^2,15^. Tankersley et al.^1^ also mention Shoemaker-Levy 9, but the comet-shaped earthwork does not show any evidence for comet fragmentation; large fragments result from rare close encounters with planets, small fragments ejected by normal comet activity are too small (~ 1 m) to cause a memorable event.

(iii) Do other cited narratives pertain to an airburst?

Practically all communities worldwide observed various celestial phenomena, also transmitted as narratives^16^, all very valuable, sometimes complementary^10^. Undated stories from various indigenous American communities are cited^1^, but e.g. the “day when the sun fell from the sky”^1^ could refer to a total solar eclipse. Then, “a black cloud rolled across the sky and was destroyed with a fiery dart by Hehnoh”^1^ citing Spencer (1909), where we read: “When the snake man went into the water in pursuit, the Black Cloud rolled across the sky, and Heh-noh slew him with a fiery dart’^17^; the whole story would need to be explained, but no element can convincingly be interpreted as airburst or its progenitor. Further, a certain word of the Shawnee (Ohio), namely *Tekoomsē* (modern transliteration) would refer “to a comet known as the Sky Panther”^1^ citing Howard^18^, who does not mention comets explicitly: “it is believed that the meteors, called shooting stars, are being fleeing from the wrath of some adversary, or from some anticipated danger. A meteor is also conceptualized by the Shawnees as a great crouching panther, hence the name of the great nineteenth century chief Tecumseh (Tekamthe) … sometimes translated as `Crouching Panther’, and sometimes as `Shooting Star’ …”^18^. If *Tekoomsē* was named after the comet of CE 1769, shortly after his birth, and if “Tecumseh’s comet”^19^ of CE 1811 was considered a positive portent^19^, this would not point to a devastating airburst in the past. There is no evidence that those various, hardly datable oral narratives would pertain to just one single, celestial event, nor that all are ~ 1700 yr old. Artistic images called “sky panther”, found onsite, are very different in nature.

Even in clearly sky-related historical transmissions, e.g. from Chinese court astronomers, it can be difficult to decide whether the record pertains to a comet or a bolide; see the report of CE 188 above: comet or fireball? This is even more challenging for narratives^16^. We recommend historical–critical methods, close reading etc.^20^ E.g., what qualifies a “horned serpent”^1^ as airbursting comet compared to an airbursting asteroid or, e.g., an auroral or halo display?
